# Hyperactivated stallion spermatozoa fail to exhibit a rheotaxis-like behaviour, unlike other species

**DOI:** 10.1038/s41598-018-34973-9

**Published:** 2018-11-15

**Authors:** Jon Romero-Aguirregomezcorta, Emer Sugrue, Lucía Martínez-Fresneda, David Newport, Sean Fair

**Affiliations:** 1 Laboratory of Animal Reproduction, Department of Biological Sciences, School of Natural Sciences, Faculty of Science and Engineering, University of Limerick, Limerick, Ireland; 2 Bernal Institute, School of Engineering, University of Limerick, Limerick, Ireland

## Abstract

The journey of spermatozoa through the female genital tract is facilitated by rheotaxis, or the cell’s preference to swim against a flow, as well as thigmotaxis, the wall tracking behaviour, which guides them to the site of fertilisation. The aim of this study was to characterise the rheotactic and thigmotactic response of stallion sperm within a microfluidic channel. Stallion sperm rheotaxis was assessed within the microfluidic channel with regard to: (i) A range of flow velocities, (ii) Varying media viscosity and (iii) Sperm hyperactivation. Sperm distribution across the microfluidic channel was also studied and compared to human and ram sperm. Stallion sperm progressed furthest at a velocity range of 10–30 µm/s, with an optimum velocity of 20 µm/s. A flow viscosity of 2.5cP or greater reduced sperm rheotaxis (P < 0.05). Stallion sperm that were hyperactivated were unable to exhibit rheotaxis within the microfluidic channel, whereas, both hyperactivated human and ram sperm did exhibit positive rheotaxis under the same conditions. The number of sperm swimming near the microfluidic channel walls was higher than in the microfluidic channel centre (P < 0.05). This is the first study to illustrate that stallion sperm are rheotactically responsive and increasing viscosity reduces this response. We also demonstrated that sperm are predominantly inclined to swim along a surface and uniquely, hyperactivated stallion sperm are non-progressive and do not exhibit a rheotactic response unlike other species.

## Introduction

Sperm have to navigate their way from the site of deposition in the lower reproductive tract to the site of fertilisation in the ampulla of the oviduct^1^. While the location of semen deposition is species dependent, as well as varying between natural mating and artificial insemination, sperm have to make the tortuous journey of in excess of 1000-fold their length to the ampulla^2^. Their journey is facilitated by the increased contractility of the reproductive tract during the follicular phase of the female cycle primarily under the control of oestrogens from the dominant follicle^3^. This contractility also stimulates mucus secretion which flows towards the cervix and results in the retrograde flow of sperm^4^. Mullins and Saacke^5^ proposed that sperm avoid retrograde flow by navigating their way through privileged pathways along the epithelial lining of the crypts and folds of the reproductive tract and more recently, a number of mechanisms have been proposed which direct sperm towards the oviducts^6,7^, namely; chemotaxis^8,9^, thermotaxis^10,11^, rheotaxis^2,4^ and thigmotaxis^12,13^.

Chemotaxis is a phenomenon whereby sperm are attracted by chemoattractants^14^ (amino acids, peptides, lipids, sulfated steroids) that have been shown to be secreted in the follicular fluid^15,16^ and by the cumulus oocyte complex^17^. Since only a fraction of capacitated human sperm (2–10%) have been shown to be chemotactically responsive^15^ it is likely that the main function of chemotaxis is the selective attraction of capacitated sperm within the ampulla by the matured oocyte^6,18^. Similarly, the temperature difference between the sperm reservoirs site in the isthmus and the warmer site of fertilisation in the ampulla (approximately +2 °C in the rabbit)^19^ has been reported to stimulate thermotactic behaviour of sperm. Thermotaxis has been reported to be mediated by calcium-permeable temperature-sensitive cation channels in human sperm^20^, however, Bahat, *et al*.^21^ reported that only 3–5% of human sperm and 7–17% of rabbit sperm were able to sense this temperature difference *in vitro* and respond to it by thermotaxis. These figures have been recently further reduced by Perez-Cerezales, *et al*.^22^, who reported that less than 1% of both human and mouse spermatozoa were selected by an *in vitro* thermotaxis system. Furthermore, they showed a significantly enriched rhodopsin labelling, a sperm thermosensor^23^, in the spermatozoa selected by thermotaxis. Thus, it appears that only a relatively small proportion of sperm respond to both chemotaxis and thermotaxis and these mechanisms are likely to be only effective in attracting sperm over short distances to the matured oocyte once sperm are in the oviducts.

Rheotaxis is the ability of organisms to orientate and swim against the flow of fluid and was proposed by Miki and Clapham^4^ as a major determinant of sperm guidance over long distances in the mammalian female reproductive tract. They demonstrated rheotactic behaviour in both capacitated and uncapacitated mouse and human spermatozoa in low and high viscosity media. Tung, *et al*.^24^ studied the migration of bull sperm against fluid flow in a microfluidic device that recreated the biophysical environment of mammalian sperm with grooves embedded on a microfluidic channel surface. They reported that microgrooves allow sperm to swim faster and more efficiently in the presence of the flow which suggests that the microgrooves present along the female reproductive tract have evolved, in part at least, to facilitate sperm progression. Similarly, Kantsler, *et al*.^2^ reported that both human and bull sperm not only swim against the flow, but swim upstream in spiral-shaped trajectories along the walls of a cylindrical microfluidic channel, and reported the ability of sperm to reverse their swimming direction upon flow reversal. The same group also assessed the effect of different shear rates (0.2–9 s^−1^) and viscosities (1–20cP) on sperm rheotactic behaviour in microfluidic channels and found that sperm upstream velocity decreased more strongly with higher viscosities for bull than for human sperm. They suggested that the difference in swimming behaviour could be due to differences in head shape; bull sperm have a flatter head than human sperm which likely suppresses the rotational motion of the cell at high viscosities, thus leading to a smaller vertical beat amplitude. El-Sherry, *et al*.^25^ reported a rheotactic response in 80–84% of bull sperm (except for very low flow velocities) and found shear stress, which is an indicator of velocity distribution, to play a critical role in regulating rheotactic behaviour of sperm. They also found that sperm tended to follow microfluidic channel walls, and those moving along the wall moved upstream faster than those swimming along the microfluidic channel centreline. Sperm swimming along the walls tended to enter side pockets without any chemical binding. This tendency of motile cells to remain close to walls and swim along boundaries follows the principles of thigmotaxis^13^.

Throughout this journey to the site of fertilisation, mammalian sperm experience biochemical changes in the form of capacitation. Hyperactivation is part of capacitation and is characterised by a high amplitude, asymmetrical beating pattern of the sperm tail manifested by a figure of eight frenzied movement pattern when viewed microscopically under a coverslip in aqueous media^26^. *In vivo*, hyperactivation has been proposed as a mechanism which is advantageous as sperm encounter viscous oviductal fluid and the viscoelastic cumulus matrix^27^ as well as aiding in detachment from oviductal epithelial cells and penetration through the cumulus cells surrounding the oocyte. However, the influence of hyperactivation on stallion sperm rheotaxis and thigmotaxis behaviour has not been reported. Moreover, to the authors’ knowledge there are no previous published studies related to stallion sperm guiding mechanisms. In this study, microfluidics was used as a tool to study stallion sperm rheotaxis in both hyperactivated and non-hyperactivated states under the influence of differing flow velocities and media viscosities and compared with human and ram sperm.

## Results

### Experiment 1: Effects of flow velocity on rheotaxis of frozen-thawed stallion spermatozoa

This experiment was performed to determine if frozen-thawed stallion sperm exhibited rheotaxis and if so establish the optimum flow velocity for maximum rheotaxis.

**Figure 1:**
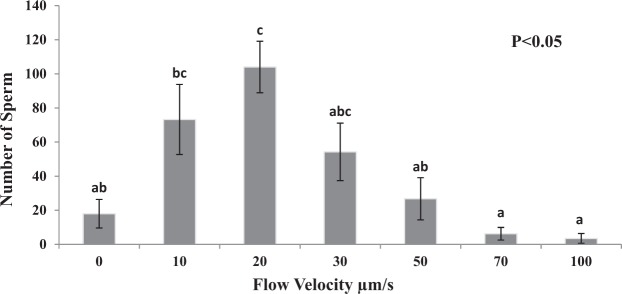
Number of stallion sperm progressing passed 15 mm in a microfluidic channel after 10 mins at varying flow velocities in tyrosine, albumin, lactate and pyruvate media (TALP; 0.9cP; Experiment 1). Vertical bars represent s.e.m. (n = 4 replicates). ^abc^Differing superscripts differ significantly (P < 0.05).

After loading of the sample, stallion sperm had to find the microfluidic channel inlet and swim into it. During this initial period, the swimming pattern was uncoordinated, but once in the microfluidic channel, sperm quickly responded to the flow and orientated against it (see Supplementary Video S1). When no flow was introduced at the inlet, sperm continued to swim in an uncoordinated pattern. There was an effect of flow velocity on sperm progression (P < 0.05; Fig. 1) as stallion sperm required a mean flow velocity of at least 10 µm/s to initiate rheotaxis. The optimum flow velocity was between 10–30 µm/s (P < 0.05) and peaked at 20 µm/s, and for this reason a velocity of 20 µm/s was chosen for all subsequent experiments. Sperm progression decreased when sperm were exposed to a flow of 50 µm/s or greater (P < 0.05) as sperm found it more difficult to swim against a faster flow.

### Experiment 2: Effect of media viscosity on frozen-thawed stallion sperm swimming patterns, rheotaxis and thigmotaxis

**Figure 2:**
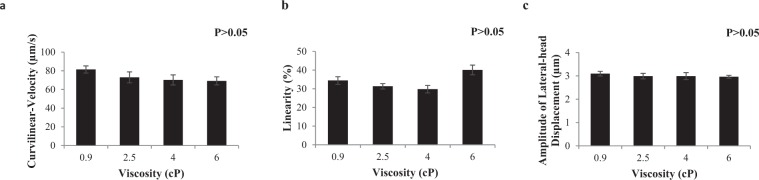
Kinematic parameters for frozen-thawed stallion sperm within a static chamber in a solution of varying viscosities (Experiment 2a). (**a**) Curvilinear-Velocity (VCL; µm/s); (**b**) Linearity (LIN, %); (**c**) Amplitude of Lateral Head Displacement (ALH; µm). Vertical bars represent s.e.m. (n = 5 replicates).

**Figure 3:**
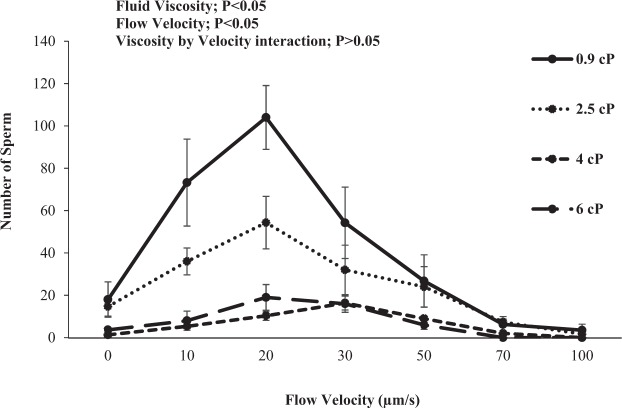
Number of frozen-thawed stallion sperm progressing passed 15 mm in a microfluidic channel after 10 mins against differing media viscosities at varying flow velocities (0–100 µm/s; Experiment 2b). Vertical bars represent the s.e.m. (n = 3 replicates).

**Figure 4:**
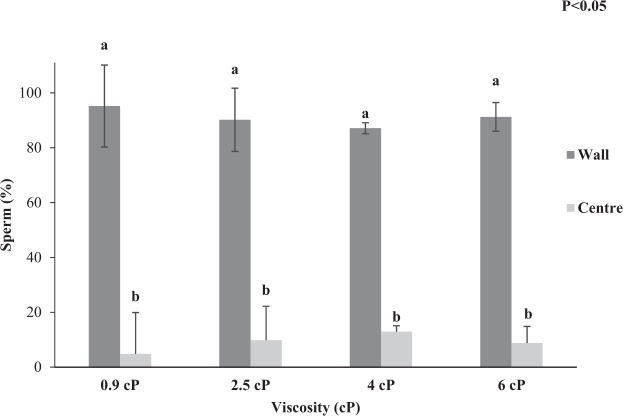
The percentage of sperm swimming at the microfluidic channel wall or in the centre position (at 15 mm along the micro-channel) in a range of viscosities (0.9–6cP; Experiment 2c). Vertical bars represent the s.e.m. (n = 3 replicates). ^ab^Differing subscripts differ significantly within viscosity (P < 0.05).

This experiment was performed to test the hypothesis that frozen-thawed stallion sperm swimming patterns and rheotaxis response is affected by fluid viscosity. In Fig. 2 it can be observed that stallion spermatozoa swimming pattern in a static drop was not affected by viscosity (P > 0.05). Although sperm kinematics were not affected by the range of viscosities assessed in a static droplet, sperm progression in the microfluidic channel was affected by the change in media viscosity (P < 0.05; Fig. 3). In addition, once exposed to a flow in the microfluidic channel sperm orientated and swam against the flow, their rate of progression was affected by the flow velocity (P < 0.05) but there was no viscosity by flow velocity interaction (P > 0.05). The optimum flow velocity for sperm progression remained the same as experiment 1 (20 µm/s) for the range of viscosity tested (0.9 to 6cP; Fig. 3). However, viscosity affected progression with the two highest viscosities (4 and 6cP) resulting in the lowest number of sperm exhibiting a rheotactic response and the biggest decrease in rheotaxis occurred between 0.9–2.5cP (Fig. 3). Sperm thigmotactic behaviour was evident, with a higher percentage of sperm swimming next to the walls than in the microfluidic channel centre (P < 0.05; Fig. 4; Supplementary Video S1). There was no effect of media viscosity (0.9, 2.5, 4 and 6cP; P > 0.05; Fig. 4) or flow velocity (0–100 µm/s; P > 0.05) on the percentage of sperm displaying wall tracking behaviour (thigmotaxis, within the microfluidic channel).

### Experiment 3: Effect of hyperactivation on rheotaxis of fresh and frozen-thawed stallion spermatozoa

This experiment was performed to assess whether hyperactivated stallion sperm had a differential rheotactic response to non-hyperactivated sperm, but also to validate the earlier results with frozen-thawed stallion sperm samples by comparing them with fresh stallion spermatozoa.

There was low motility and progressive motility in the frozen-thawed samples, however when compared, the kinematic parameters between fresh and frozen-thawed, either treated with procaine or not, they are similar (Table 1; Supplementary Videos S2–S5 for non-hyperactivated and hyperactivated, frozen-thawed and fresh spermatozoa, respectively). Moreover, the effect of inducing hyperactivation with 5 mM procaine was similar in fresh and frozen-thawed samples, with statistically significant increases observed in VCL and ALH; and decreases in VSL, LIN, STR and WOB (P < 0.05). The results for VCL, ALH and LIN are consistent with hyperactivation parameters previously described in the literature (reviewed by Hinrichs and Loux^28^). Swimming pattern of stallion sperm hyperactivated using procaine was confirmed to be the same as when the sperm were hyperactivated by increasing the pH.

**Table 1:** Motility and kinematic parameters of fresh and frozen-thawed stallion spermatozoa which were incubated for 15 min either in the presence of 5 mM procaine or not (control) and evaluated by computer assisted sperm analysis (CASA) in a static droplet.

Sample	Group	Mot	Prog	VCL	VSL	VAP	LIN	STR	WOB	ALH	BCF
Fresh	Control	79.2 ± 4.0	55.5 ± 3.6	88.2 ± 5.3	25.1 ± 2.7	65.3 ± 4.3	32.6 ± 1.7	44.9 ± 1.5	73.7 ± 2.0	2.9 ± 0.1	5.6 ± 0.4
	5 mM Procaine	78.5 ± 5.4	55.1 ± 5.1	112.9 ± 6.7*	16.9 ± 1.0*	64.4 ± 1.7	20.7 ± 1.0*	34.8 ± 1.8*	58.5 ± 0.9*	4.4 ± 0.3*	5.8 ± 0.3
Frozen	Control	32.1 ± 4.6	19.5 ± 4.5	82.9 ± 8.7	28.5 ± 4.4	64.7 ± 7.5	34.1 ± 2.1	48.0 ± 2.2	70.4 ± 2.7	2.8 ± 0.2	4.1 ± 0.7
	5 mM Procaine	35.9 ± 5.9	26.0 ± 4.6	110.7 ± 4.2*	15.0 ± 0.8*	56.1 ± 0.3	16.5 ± 0.6*	31.6 ± 1.1*	50.6 ± 1.3*	4.8 ± 0.4*	7.5 ± 1.1

Fresh or frozen-thawed stallion spermatozoa were incubated in TALP medium for 15 min at pH 7.4 in presence or absence of 5 mM procaine and motility parameters were measured by CASA. Three replicates were performed. Results are expressed as mean ± SEM. The CASA derived motility and kinematic parameters assessed were motility (Mot, %), progressive motility (Prog, %), curvilinear velocity (VCL; µm/s), straight line velocity (VSL; µm/s), average path velocity (VAP; µm/s), linearity (LIN, %), straightness (STR, %), wobble (WOB, %), amplitude of the lateral head displacement (ALH; µm) and beat cross frequency (BCF; Hz). Asterisks indicate statistical significance within column in either fresh or frozen-thawed semen samples (P < 0.05).

On the other hand, when exposed to a fluid flow, both fresh and frozen-thawed stallion sperm which had been incubated in the presence of 5 mM procaine, had reduced progression compared to the control treatment which had high levels of progression (see Supplementary Videos S1, S6–S8 for non-hyperactivated and hyperactivated, frozen-thawed and fresh spermatozoa, respectively). This result was confirmed by the observation of stallion sperm samples that were pre-loaded into the microfluidic channel, it was obvious from these that the hyperactivated stallion sperm did not exhibit rheotaxis (see Supplementary Videos S6 and S8 for hyperactivated frozen-thawed and fresh spermatozoa, respectively).

### Experiment 4. Comparison of the swimming patterns and rheotactic response of hyperactivated frozen-thawed stallion, ram and human spermatozoa

This experiment was performed to investigate if hyperactivated sperm from other species (human and ram) behaved in a similar manner to hyperactivated stallion sperm.

**Figure 5:**
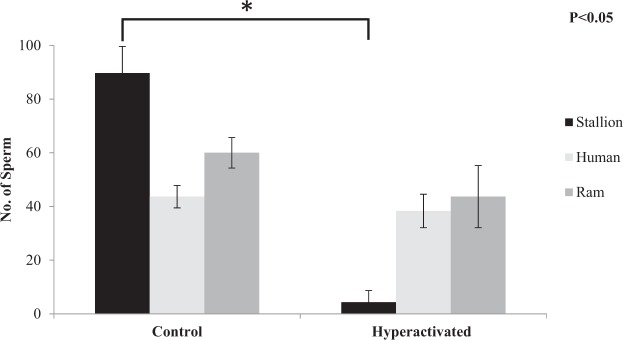
Number of stallion, human and ram sperm progressing passed 15 mm in a microfluidic channel after 10 mins against a flow (20 µm/sec) following treatment with hyperactivation agonists or not (Control; Experiment 4). Vertical bars represent the s.e.m. (n = 3 replicates). *Represents statistical significance.

**Figure 6:**
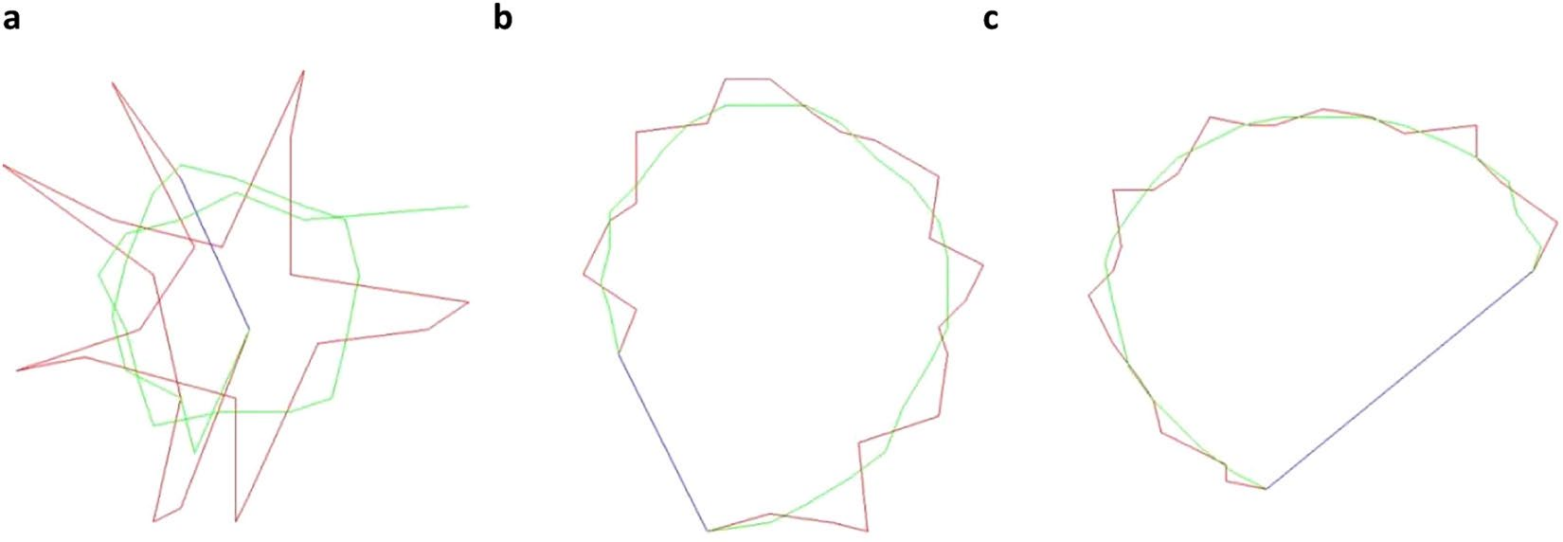
Computer assisted sperm analysis showing the swimming patterns of hyperactivated stallion (**a**), human (**b**) and ram (**c**) sperm (Experiment 3). The different coloured lines represent the curvilinear velocity (VCL, red), average path velocity (VAP, green) and straight line velocity (VSL, blue).

Incubation of frozen-thawed stallion, human and ram sperm with the hyperactivation agonists, procaine, progesterone and caffeine, respectively, increased the percentage of hyperactivated sperm compared to the control treatment (P < 0.05; Supplementary Figure S9 and Videos S2 and S3), as characterised by high-amplitude flagellar waves and asymmetrical flagellar beating and assessed subjectively in a static non-viscous droplet. Following this the number of stallion, human and ram sperm that progressed passed 15 mm after 10 min in the microfluidic channel, in both hyperactivated and non-hyperactivated samples was assessed. Interestingly, despite the reduced progression of hyperactivated stallion sperm, hyperactivated human and ram sperm exhibited rheotaxis, showing clear differences between species (Fig. 5). There was a distinct difference in hyperactivated swimming patterns of the three species (Fig. 6) represented by a unique star shaped swimming pattern of the stallion sperm when hyperactivated, in comparison to the circular pattern of the human and ram sperm.

## Discussion

To navigate their way towards the site of fertilisation in the oviducts, sperm orientate and swim against a flow of mucus, a phenomenon known as rheotaxis^4,7^ and while doing so, are guided by the sensation of touch along the epithelial lining of the reproductive tract (thigmotaxis)^12,13^. Both rheotaxis and thigmotaxis have been shown in a number of species, with evidence suggesting that rheotaxis in particular, is a major factor in long distance sperm guidance^4^. However, the role of these responses in guiding stallion sperm towards the site of fertilisation and the effect of viscosity and flow velocity on them has not yet been investigated or characterised in stallion sperm.

This study offers a novel insight into stallion sperm rheotaxis and how it is influenced by viscosity and associated species differences in the ability of hyperactivated sperm to exhibit rheotaxis. It demonstrated (i) that stallion sperm respond to a fluid flow and also that their rheotactic response is affected by fluid viscosity (ii) it clearly illustrates for the first time that hyperactivated stallion sperm do not exhibit a rheotaxis-like behaviour and that their swimming pattern against a fluid flow is uncoordinated and non-progressive, similar to the erratic pattern traditionally observed in a static environment. In contrast, this study showed (iii) that both hyperactivated human and ram sperm do indeed display positive a rheotaxis-like behaviour, however, hyperactivated motility did not affect their rate of progression as it does in bull sperm^29^.

Although rheotaxis determination was not validated by a directionality method such as videomicroscopy, as described in other species^4,30,31^, this study has clearly shown that stallion sperm actively respond and adapt to fluid flow similar to human^4^ and bull^29^ and is in agreement with reported differences in the percentage of sperm exhibiting rheotaxis at different flow velocities in a microfluidic channel^25^. The only known published data on the rate of fluid flow *in vivo* is that by Miki and Clapham^4^ who found the velocity of fluid flow within the oviduct of the mouse to be approximately 18 µm/s. This is similar to the current study which found the optimum flow velocity for maximum stallion sperm rheotaxis to be 20 µm/s. In addition to fluid flow sperm must also overcome mucus of varying viscosity enroute to the site of fertilisation, and Coy, *et al*.^32^ proposed that the viscosity of the reproductive secretions reduced the sperm to oocyte ratio thereby reducing polyspermy. The changes in oviductal fluid composition, under hormonal influences provide the optimum conditions for sperm transport, fertilisation and early embryo development^33^, with oviductal fluid viscosity in the peri-ovulatory period estimated to be as low as 2–3cP^4^. The results of the current study demonstrate that sperm progression was affected by the media viscosity within the range 0.9–6cP. It was not possible to pump higher viscosities through the microfluidic channel, and although the viscosities were relatively low they were sufficient to demonstrate that the viscosity of the reproductive secretions does indeed have an effect on sperm rheotaxis. This also supports the hypothesis that fluctuations in the oviductal fluid viscosity during the oestrus cycle can contribute to reducing the number of sperm at the site of fertilisation^32^.

A study by Kantsler, *et al*.^2^ reported changes in the flagellar beat pattern of human sperm in a static droplet, from a strong helical beat component in a low viscosity fluid (3cP) to a more planar wave forms in a high viscosity fluid (20cP). The same study reported differences in the beat component between human and bull sperm, which was explained by the differences in head shape, since bull sperm have a flatter head than human sperm. Therefore, the study suggested sperm alter their rheotactic swimming patterns but not their rate of progression in viscous fluid. This is in disagreement with the findings of this study, where there was a significant reduction in sperm progression with increasing viscosity.

Hyperactivation is a key step in the capacitation process and has long been proposed as a process by which sperm detach from the epithelial lining of the sperm reservoir in the isthmus, and as a means to penetrate the oocyte cumulus complex and zona pellucida^34,35^. In support of this, Ho, *et al*.^34^ showed that mice sperm unable to hyperactivate were subsequently incapable of unbinding from the oviductal epithelium. Interestingly, this study found that hyperactivated stallion sperm unable to progress against a flow within a microchannel. Miki and Clapham^4^ proposed that as viscosity increases the swimming pattern of capacitated mouse sperm becomes more direct, while, Perez-Cerezales, *et al*.^7^ reported that rheotaxis is dependent on hyperactivation. This contrasts with the findings of this current study which showed that hyperactivation clearly inhibited stallion sperm rheotaxis. Interestingly, human and ram sperm did display rheotaxis when hyperactivated, similar to results observed with bull sperm as described by Kantsler, *et al*.^2^ and Johnson, *et al*.^29^. The rheotactic response of ram and human sperm was not significantly altered by hyperactivation unlike the findings of Johnson, *et al*.^29^ who reported greater progression of hyperactivated bull sperm. As spermatozoa of the three species displayed rheotaxis when not hyperactive, it suggests that stallion sperm hyperactivation is somehow different in function to the other species examined. It has also been reported that rheotaxis, thermotaxis and chemotaxis are only effective on capacitated sperm (human and mouse)^7^, however, this was not the case in this current study, which showed that both human and stallion sperm displayed rheotaxis irrespective their capacitation status. To rule out the possibility that the hyperactivated stallion sperm were unable to find their way into the microfluidic channel due to the erratic swimming pattern, sperm were carefully pre-loaded into the microfluidic channel where they were exposed to a flow. The same result was observed, whereby, the hyperactivated stallion sperm were non-progressive, unable to display rheotaxis and were swept downstream with the flow (see Supplementary Video S6).

Loux, *et al*.^36^, demonstrated that the sperm-specific pH-gated calcium channel (CatSper) is present in equine sperm. CatSper are weakly voltage-dependent, Ca^2+^ selective, and pH-sensitive ion channels that control the entry of Ca^2+^ ions into the spermatozoa. In mice, those spermatozoa lacking CatSper were unable to hyperactivate^34^. On the contrary, in equine species, procaine (5 mM), which has been widely used to induce hyperactivation in spermatozoa^36–38^, inducing the greatest degree of hyperactivation when compared to high-pH medium and 4 mM 4-aminopyridine, does not appear to act via CatSper^36^ but by inhibiting sodium influx^39^. Since hyperactivation is likely to be required for normal fertilisation^40^, these studies have illustrated the importance to determine the mechanism by which procaine induces hyperactivated motility in equine spermatozoa. In this regard, we have demonstrated the lack of upstream progression of the stallion spermatozoa after the induction of hyperactivation with 5 mM procaine. We have added valuable information relating to equine sperm behaviour *in vitro* under a rheotactic challenge, in an attempt to mimic the sperm upstream progression against the downstream fluid flow in the mare genital tract.

The inability of hyperactivated stallion sperm to exhibit rheotaxis when exposed to a flow, or when preloaded into the microfluidic channel illustrates clear species difference in rheotaxis. This could possibly, be explained by the unique star shaped swimming pattern of the stallion sperm when hyperactivated, in comparison to the circular like swimming pattern of the human and ram sperm (Fig. 6). The swimming pattern remained the same irrespective of whether the stallion sperm were hyperactivated with procaine or by using a more physiological strategy of increasing the extracellular pH. Commonly used motility parameters that define hyperactivation include but are not limited to; an increase in curvilinear velocity and amplitude of lateral head displacement and a decrease in linearity (reviewed by Hinrichs and Loux^28^). These changes were observed in the hyperactivated stallion sperm in this study and further support the finding that the hyperactived motility, induced using procaine, is representative of physiological hyperactivation. It is likely that the variation in the swimming patterns is due to the different head shapes of the three species. This illustrates that, when viewed within the confines of a static low viscous saline fluid, hyperactivated stallion sperm have a vigorous, erratic and non-progressive movement pattern^27^, and this was not affected by exposure to a flow during rheotaxis within a microfluidic channel. However, hyperactivated human and ram sperm appear to develop a more progressive swimming pattern when exposed to a flow similar to the bull^29^. These species differences are particularly interesting as they raise questions over the function of hyperactivated motility in stallion sperm. In particular, the previous studies which suggest that the purpose of hyperactivated motility is to aid the sperm in detaching from the reservoirs and penetration of the viscous oviductal mucus^35,41^. The non-progressive swimming pattern of the hyperactivated stallion sperm when exposed to a flow suggest that it is unlikely that the sperm would be able to swim to the site of fertilisation in the ampulla. One possible explanation is a model proposed by Armon and Eisenbach^41^, who suggested that sperm behaviour was affected by a chemoattractant gradient and that capacitated sperm maintain their course of swimming, specifically, they swim straighter and repress hyperactivated motility (turn less) when swimming up a concentration gradient. They suggested that sperm in the presence of a chemoattractant are continuously stimulated and this results in the sperm swimming straight without turns or hyperactivation events and a decrease in the concentration gradient increases the frequency of these hyperactivation events. Furthermore, they suggested that the function of hyperactivation is to re-orientate the sperm towards the concentration gradient through sharp turning. It is possible that chemotaxis and not rheotaxis would influence the hyperactivated stallion sperm to swim straighter and therefore, guide the sperm to the oocyte. Chemotaxis is thought to be a short range guidance mechanism occurring within in the order of millimeters within the oviduct, however, Bian, *et al*.^42^ and Armon and Eisenbach^41^ reported that in mice a gradient of natriuretic peptide precursor A (NPPA; a known chemoattractant *in vitro*) was found as far as the utero-tubule junction, raising the possibility that chemotaxis has a longer range of sperm guidance than previously believed. Therefore, we propose that rheotaxis is central to directing sperm to the oviducts in all mammalian species but there are species-specific mechanisms guiding hyperactivated sperm towards the oocyte. In horses, the biophysical role in long-distance guidance is demonstrated, but assuming hyperactivation does actually occur *in vivo* in horses, the synergystic effect with the other taxis, i.e. chemotaxis and thermotaxis, should be taken into acount when attempting to explain the *in vivo* sperm guiding mechanisms to the site of fertilisation.

Sperm thigmotactic behaviour was evident in the non-hyperactivated stallion sperm, and in both hyperactivated and non hyperactivated human and ram sperm. This suggests that non-hyperactivated stallion sperm and both hyperactivated and non-hyperactivated human and ram sperm have a preference to move against a flow^24^ and along surfaces^13,43^. This mimics the privileged pathways of the cervix and uterus, perhaps in search of the epithelial lining of the utero-tubular junction and isthmus where they can bind and form a sperm reservoir. Indeed, non-capacitated sperm have a greater affinity to bind to the oviductal epithelium but as sperm complete capacitation^44^, and hyperactivate, this aids in them pulling away from the epithelium. Current methods of sperm analysis in media in which the conditions such as viscosity and temperature remain constant are useful, however, they fail to take into account the complex and varied conditions encountered by the sperm along the female reproductive tract^45^. This was also the sentiment of Kirkman-Brown and Smith^46^ who cautioned against making conclusions about sperm motility based on single factors without taking into consideration all the other elements that may be at play.

To the best of the authors’ knowledge, this is the first published study to characterise stallion sperm rheotaxis. The results of this study clearly demonstrate that stallion sperm rheotaxis-like response varies with flow velocity and is reduced by increasing fluid viscosity. It also established that hyperactivated stallion sperm swim in an uncoordinated star shaped pattern and they are not rheotactically responsive when exposed to a flow or viscous media, unlike human and ram sperm. Thigmotaxis was confirmed by a clear preference of sperm to swim against the flow next to the walls of the microfluidic channel. In conclusion, the study of sperm in microfluidic channels is a useful tool to increase our understanding of how sperm behave in the female reproductive tract and facilitates the evaluation of sperm rheotactic and thigmotactic behaviour in response to varying physiological conditions.

## Material and Methods

### Reagents

All chemicals and reagents were purchased from Sigma-Aldrich Ireland Ltd (Arklow, Ireland) unless otherwise stated.

### Sample Preparation

Frozen stallion sperm from three Irish Sport Horse Stallions was donated from a commercial stud. Thawed sperm (37 °C for 30 seconds) was pooled to reduce inter-male variation in all experiments. Post-thaw sperm motility of the pooled sample was assessed subjectively using a phase-contrast microscope (BX60; Olympus, Centre Valley, USA) as a quality control check and only samples with greater than 30% total motility were used for experiments. Preliminary work demonstrated that a concentration of 20 × 10^6^ sperm/mL was the optimum for use in the microfluidic channel and therefore this was used throughout all experiments. Sperm were diluted to this concentration using Tyrode’s albumin lactate and pyruvate (TALP) media^47^ and a 10 µL sample was placed in the sample inlet. Media viscosity in all experiments was 0.9cP and flow velocity was 20 µm/s, except the experiments in which different viscosities or flow velocities were assessed. The viscosity of TALP was assessed using a viscometer (Brookfield, DV2T, Lab Unlimited, Dublin, Ireland) and was determined to be 0.9cP. Methyl cellulose (4000cP) was added to TALP, at 0.1, 0.15 and 0.2% (w/v) in order to increase the viscosity of the media to 2.5, 4 and 6cP, respectively. All viscosities were confirmed using the viscometer. Fresh stallion semen was obtained from three ejaculates collected from three different Irish Sport Horse Stallions. These samples were processed as described above for the frozen-thawed samples with the exception that they were diluted in INRA96 diluent and stored at 4 °C. Frozen human sperm samples were obtained from the Cryos International ApS Biobank (Aarhus, Denmark). Straws from three different donors were thawed and pooled. Frozen ram sperm samples from three different males, donated from a commercial breeding centre, where also thawed and pooled before each experiment. Frozen human and ram sperm were thawed and diluted as described above.

### Microfluidic Channel

A microfluidic channel manufactured from polymethyl methacrylate (PMMA), was purchased from the Microfluidic ChipShop (Jena, Germany). The microfluidic channel measured 800 µm wide, 20 µm deep and 58.5 mm in length. It was connected to a syringe pump (Standard Infuse/Withdraw PHD ULTRA™ Syringe Pump, Harvard Apparatus, Holliston, USA) by silicone tubing and a connector (Male Mini Luer fluid connector, Microfluidic ChipShop, Jena, Germany). The microfluidic channel was primed with TALP to remove air bubbles, following this, the flow was established at controlled rates of 0–100 µm/s. The sperm sample was loaded into the starting inlet of the microfluidic channel allowing sperm to orient against the oncoming flow and swim against it. The number of sperm that passed 15 mm after 10 min was used as the criteria for the assessment of sperm rheotaxis. Sperm progression was assessed under 200X magnification on an inverted microscope (CK40; Olympus, Center Valley, USA). The channel was cleaned with dH_2_O after use, and air was then pumped through to remove any excess fluid remaining in the channel.

### Computer Assisted Sperm Analysis

Computer assisted sperm analysis (CASA) was performed using the Sperm Class Analyzer system (SCA; Microptic S.L., Barcelona, Spain) to assess motility and kinematic parameters. A 5 µL drop of diluted semen was placed in a pre-warmed Leja chamber (10 µm depth; IMV Technologies, L’Aigle, France) and analysed for sperm motion characteristics using factory CASA stallion settings. A minimum of five randomly selected microscopic fields with at least 200 sperm were analysed in each sample using a phase contrast microscope (BX60; Olympus, Centre Valley, USA) with a fitted heated stage at 37 °C. A manual correction was carried out to add or delete sperm or debris as required. The CASA derived motility and kinematic parameters assessed were motility, progressive motility (sperm which display a forward progressive linear movement), straight line velocity (VSL; µm/s; the time averaged velocity of a sperm along a straight line from its first position to its last position), average path velocity (VAP; µm/s; the time averaged velocity of a sperm along its average path), curvilinear velocity (VCL; µm/s; the time averaged velocity of the sperm along its actual path), amplitude of the lateral head displacement (ALH; µm; the width of the lateral movement of the sperm head about its average path) linearity (LIN%; VSL/VCL), straightness (STR%; VSL/VAP), and beat cross frequency (BCF; Hz; the number of times the sperm head crosses the direction of movement per second or the average rate at which the curvilinear path crosses the average path). Spermatozoa with a VAP less than 10 µm/s were considered non-motile.

### Ethics

All studies were carried out in accordance with the University of Limerick approved guidelines. Sperm from animal species were donated from commercial animal breeding centres while human sperm was obtained from the Cryos International ApS Biobank (Aarhus, Denmark). All donors gave informed consent and the use of human sperm samples for research was approved by Faculty of Sciences and Engineering Research Ethics Committee at the University of Limerick.

## Experimental Design

### Experiment 1: Effects of flow velocity on rheotaxis of frozen-thawed stallion spermatozoa

The aim of this experiment was to determine if frozen-thawed stallion sperm exhibited rheotaxis and if so establish the optimum flow velocity for maximum rheotaxis. The sperm rheotactic response was assessed in 7 different velocities, namely 0, 10, 20, 30, 50, 70 and 100 µm/s, as described above. Four replicates were completed.

### Experiment 2: Effect of media viscosity on frozen-thawed stallion sperm swimming patterns, rheotaxis and thigmotaxis

This experiment tested the hypothesis that frozen-thawed stallion sperm swimming patterns and rheotaxis response is affected by fluid viscosity. The first objective was to determine the effect of media viscosity on stallion sperm swimming patterns in a static droplet. Frozen-thawed stallion sperm were diluted to a concentration of 20 × 10^6^ sperm/mL in different viscosities (0.9, 2.5, 4, 6cP), incubated for 15 mins at 37 °C and assessed for motility and kinematic parameters in a static environment within a pre-warmed Leja chamber (10 µm depth; IMV Technologies, L’Aigle, France), using CASA. Five replicates were completed.

The second objective was to assess stallion sperm progression within the microfluidic channel by creating a flow with media of different viscosities, and to determine if the viscosity affected the optimum flow velocity for maximum sperm rheotaxis. The sperm rheotactic response was assessed in 7 different velocities, namely 0, 10, 20, 30, 50, 70 and 100 µm/s, each in media of four different viscosities (0.9, 2.5, 4 and 6cP) as described previously. Three replicates were completed. It was not possible to pump higher viscosities through the microfluidic channel.

The third objective was to assess the effect of viscosity on the stallion sperm distribution across the microfluidic channel in order to quantify sperm wall tracking behaviour (thigmotaxis). Sperm that were swimming touching the walls of the microfluidic channel were considered as sperm at the wall and the remainder of sperm that were not in contact with the wall were considered as sperm in the centre of the microfluidic channel. Three replicates were completed.

### Experiment 3: Effect of hyperactivation on rheotaxis of fresh and frozen-thawed stallion spermatozoa

The objective of this experiment was to assess if hyperactivated stallion sperm had a differential rheotactic response to non-hyperactivated sperm. However, as all the previous experiments were performed with frozen-thawed stallion spermatozoa, we aimed to validate the use of frozen-thawed samples by comparing them with fresh stallion spermatozoa.

First, fresh and frozen-thawed stallion sperm were incubated for 15 min either in the presence of 5 mM procaine, a known stallion sperm hyperactivation agonist^36^, or not (control) and evaluated by CASA in a static droplet to assess motility and kinematic parameters and the swimming pattern compared to that of stallion sperm hyperactivated by incubation in media of pH = 8.0. pH was adjusted to 8.0 by adding 1N NaOH. Three replicates were completed.

Second, to assess the effect of hyperactivation on rheotaxis and to determine whether the rheotactic response was the same for both hyperactivated and non-hyperactivated fresh and frozen-thawed samples, samples where incubated as above for 15 min prior to loading them in the microfluidic channel. Following this, stallion sperm rheotactic response for both hyperactivated and non-hyperactivated samples was assessed when exposed to a flow velocity of 20 µm/s. In addition to this, the samples were also pre-loaded into the microfluidic channel and further assessed for a rheotactic response. Three replicates were completed.

### Experiment 4. Comparison of the swimming patterns and rheotactic response of hyperactivated frozen-thawed stallion, ram and human spermatozoa

Based on the results of the previous experiments, a further objective was to investigate if hyperactivated sperm from other species (human and ram) behaved in a similar way to hyperactivated stallion sperm. Based on the scientific literature, progesterone (10 µM)^48^ and caffeine (10 mM)^49^ were used to hyperactivate human and ram sperm, respectively, and these were confirmed in our preliminary studies. Subsequently, the rheotactic response of hyperactivated and non hyperactivated human and ram sperm was assessed under the same experimental condition as described for stallion sperm. Three replicates were completed. Finally, the swimming trajectories of hyperactivated stallion, human and ram sperm were assessed using CASA to define species differences.

### Statistical Analysis

Data were analysed using Statistical Package for the Social Sciences (SPSS) (version 22, IBM, Chicago, USA). Data were first examined for homogeneity of variance using Levene’s test and later for normality of distribution using the Shapiro-Wilk test, transformed where appropriate and analysed using a univariate analysis of variance (ANOVA). Data from experiment 2b (stallion sperm progression within the microfluidic channel with different flow velocities and media viscosities; Fig. 3) and 2c (stallion sperm distribution across the microfluidic channel with different media viscosities; Fig. 4) were transformed using a square root transformation. The transformed data were used to calculate the P values; however, the corresponding means and standard error of the non-transformed data are presented in the results. Post hoc tests were conducted using the Bonferroni test and results were reported as the mean ± standard error of the mean (s.e.m). P < 0.05 was considered statistically significant.

## Electronic supplementary material

Supplementary Information

Supplementary Video S1.

Supplementary Video S2.

Supplementary Video S3.

Supplementary Video S4.

Supplementary Video S5.

Supplementary Video S6.

Supplementary Video S7.

Supplementary Video S8.

## Data Availability

The datasets generated during and/or analysed during the current study are available from the corresponding author on reasonable request.
