# Supplementary material for: Hyperactivated stallion spermatozoa fail to exhibit a rheotaxis-like behaviour, unlike other species
Source: Sci Rep. 2018 Nov 15;8:16897. doi: 10.1038/s41598-018-34973-9 (PMC6237856; doi:10.1038/s41598-018-34973-9)

Hyperactivated stallion spermatozoa fail to exhibit a rheotaxis-like behaviour, unlike other species

Jon Romero-Aguirregomezcorta, Emer Sugrue, Lucía Martínez-Fresneda, David Newport and Sean Fair*

Supplementary Information

Supplementary Video S1. Non-hyperactivated frozen-thawed stallion sperm in a microfluidic channel exhibiting rheotaxis. Sperm detect the flow and swim against it during rheotaxis. The arrow indicates the direction of the flow.

Supplementary Video S2. Non-hyperactivated frozen-thawed stallion sperm in a static environment. This video was recorded as a control in order to compare the movement pattern of hyperactivated and non-hyperactivated sperm.

Supplementary Video S3. Hyperactivated frozen-thawed stallion sperm in a static environment. After incubation with procaine 5 mM sperm showed high-amplitude flagellar waves and asymmetrical flagellar beating when assessed in a static droplet.

Supplementary Video S4. Non-hyperactivated fresh stallion sperm in a static environment. This video was recorded as a control in order to compare the movement pattern of hyperactivated and non-hyperactivated sperm.

Supplementary Video S5. Hyperactivated fresh stallion sperm in a static environment. After incubation with procaine 5 mM sperm showed high-amplitude flagellar waves and asymmetrical flagellar beating when assessed in a static droplet.

Supplementary Video S6. Hyperactivated frozen-thawed stallion sperm in a microfluidic channel. Stallion sperm incubated with procaine (5 mM) were non-progressive and unable to exhibit rheotaxis. The arrow indicates the direction of the flow.

Supplementary Video S7. **Non-hyperactivated fresh stallion sperm in a microfluidic channel exhibiting rheotaxis.** Sperm detect the flow and swim against it during rheotaxis. The arrow indicates the direction of the flow.

Supplementary Video S8. Hyperactivated fresh stallion sperm in a microfluidic channel. Stallion sperm incubated with procaine (5 mM) were non-progressive and unable to exhibit rheotaxis. The arrow indicates the direction of the flow.

Supplementary Figure S9. The percentage of hyperactivated sperm compared to the control treatment following incubation with the hyperactivation agonists, procaine (stallion), progesterone (human) and caffeine (ram) as characterised by high-amplitude flagellar waves and asymmetrical flagellar beating and assessed subjectively in a static saline droplet (Experiment 4). Vertical bars represent the s.e.m. (n=3 replicates). [ab]Differing superscripts differ significantly within species (P<0.05).

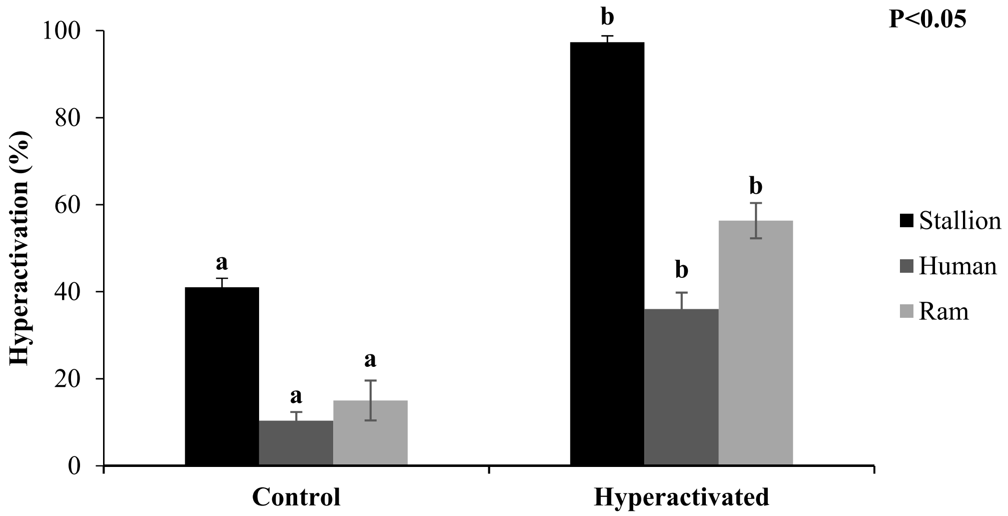

Supplement: Supplementary file 1 — Supplementary Information [file 41598_2018_34973_MOESM1_ESM.pdf]
